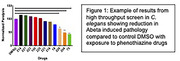# Translating high throughput screens in C elegans and microglia to humans with Medicare data to repurpose drugs to treat Alzheimer’s disease

**DOI:** 10.1002/alz.086778

**Published:** 2025-01-09

**Authors:** Rachel R Litke, Arushi Arora, Yihan Wang, Melissa D Aldridge, Fred C Ko, Sven Sandin, Mary Sano, Abraham Reichenberg, Charles V Mobbs

**Affiliations:** ^1^ Icahn School of Medicine at Mount Sinai, New York, NY USA; ^2^ Icahn School Of Medicine at Mount Sinai, New York, NY USA; ^3^ James J. Peters VA Medical Center, Bronx, NY USA

## Abstract

**Background:**

Despite increasing knowledge of the etiology of neurodegenerative diseases, translation of these benefits into therapeutic advances for Alzheimer’s Disease and related diseases (ADRD) has been slow. Drug repurposing is a promising strategy for identifying new uses for approved drugs beyond their initial indications. We developed a high‐throughput drug screening platform aimed at identifying drugs capable of reducing proteotoxicity in vivo (Aß toxicity in *Caenorhabditis elegans*) AND inhibiting microglial inflammation (TNF‐alpha IL‐6), both implicated in driving AD(figure attached with sample of results in *C. elegans*). These screens led us to prioritize 50 potentially protective FDA‐approved drugs. We propose to test our screening results in humans using administrative claims data collected from the Centers for Medicare and Medicaid Services

**Method:**

This is an observational retrospective pharmaco‐epidemiological longitudinal cohort study. The cohort is a random sample of 1,000,000 beneficiaries, aged 65‐75 years, followed for 10 consecutive years, requested from CMS. Files include MedPar, Outpatient, Carrier, Hospice to maximize inclusion of AD beneficiaries according to Bynum algorithm, and Part D event for drug prescription details. We will use Cox regression, to compute Hazard Ratios and associated 95% confidence intervals, of the association between drug exposure status and the risk of ADRD. We will examine potential confounding by indication, drug target, and competing risks.

**Result:**

1/We propose to assess if drugs which reduce Ab toxicity in a *C. elegans* model of AD AND reduce microglial inflammation reduce the risk of developing ADRD in humans, using Medicare claims. 2/We propose to assess if drugs which reduce inflammation, reduce the risk of developing ADRD in humans, using the Medicare claims.

**Conclusion:**

The end goal of the study is to identify drugs to be repurposed to treat ADRD and to accumulate strong epidemiological evidence in addition to the existing evidence from the model organism *C. elegans* and cell culture studies.